# Dynamic channel adjustments in the Jingjiang Reach of the Middle Yangtze River

**DOI:** 10.1038/srep22802

**Published:** 2016-03-11

**Authors:** Junqiang Xia, Shanshan Deng, Jinyou Lu, Quanxi Xu, Quanli Zong, Guangming Tan

**Affiliations:** 1 State Key Laboratory of Water Resources and Hydropower Engineering Science, Wuhan University, Wuhan 430072, China; 2 Changjiang River Scientific Research Institute, Wuhan 430010, China; 3 Bureau of Hydrology, Changjiang Water Resources Commission, Wuhan 430010, China

## Abstract

Significant channel adjustments have occurred in the Jingjiang Reach of the Middle Yangtze River, because of the operation of the Three Gorges Project (TGP). The Jingjiang Reach is selected as the study area, covering the Upper Jingjiang Reach (UJR) and Lower Jingjiang Reach (LJR). The reach-scale bankfull channel dimensions in the study reach were calculated annually from 2002 to 2013 by means of a reach-averaged approach and surveyed post-flood profiles at 171 sections. We find from the calculated results that: the reach-scale bankfull widths changed slightly in the UJR and LJR, with the corresponding depths increasing by 1.6 m and 1.0 m; the channel adjustments occurred mainly with respect to bankfull depth because of the construction of large-scale bank revetment works, although there were significant bank erosion processes in local regions without the bank protection engineering. The reach-scale bankfull dimensions in the UJR and LJR generally responded to the previous five-year average fluvial erosion intensity during flood seasons, with higher correlations being obtained for the depth and cross-sectional area. It is concluded that these dynamic adjustments of the channel geometry are a direct result of recent human activities such as the TGP operation.

Bankfull (channel) geometry in an alluvial river refers to the channel dimensions associated with the bankfull discharge, which is usually represented by the channel width, cross-sectional area, and the corresponding mean depth at bankfull level^1^,^2^,^3^. These bankfull dimensions are important design parameters in various river regulation works and flood control management, and therefore it is necessary to investigate the variation of these parameters in an alluvial river undergoing continuous channel degradation, such as the Jingjiang Reach of the Middle Yangtze River.

Bankfull geometry is closely associated with the concept of hydraulic geometry, and many hydraulic geometry relations have been proposed to describe the bankfull dimensions in stable or quasi-stable rivers, using empirically fitted power functions of a characteristic discharge or controlled drainage area^1^,^3^,^4^,^5^,^6^. Although the concept of bankfull was proposed earlier for equilibrium or quasi-equilibrium rivers^1^,^2^, researchers in river dynamics extended this concept to disequilibrium rivers. The extended concept has been used widely to investigate the variation in bankfull channel dimensions of the reaches below dams^7^,^8^,^9^. For example, a methodology for the prediction of bankfull area was developed based on a general delayed response equation, which accounted for the cumulative effect of previous years’ flow and sediment conditions on the channel evolution downstream of a dam^7^. Previous methods to predict the variation in bankfull geometry usually apply to a specified cross-section in a disequilibrium reach, and the obtained results can be unrepresentative of the bankfull geometry of a total reach, because there is great variability in the channel geometry along the reach^7^,^8^. Therefore, a reach-scale concept is appropriate to investigate the bankfull channel dimensions of an alluvial river, and reach-averaged variables can provide a more representative geometry and statistics characterising longitudinal variability^3^,^4^,^9^.

Human activities such as dam construction can significantly alter the natural flow and sediment regimes in alluvial rivers, which can have important consequences for variation in channel morphology^8^,^10^,^11^,^12^,^13^,^14^. For example, an exponential function was presented to describe the active channel width change for the Hwang River in South Korea, and this function was based on a hypothesis that the change in channel width is proportional to the difference between the current channel width and the equilibrium width^8^. In addition, river regulation works can also influence the adjustments in bankfull channel geometry of an alluvial river^15^,^16^,^17^,^18^,^19^. The hydrodynamic and morphodynamic responses to various river regulation works in the Lower Missouri River were investigated using a fixed-discharge analysis method^16^. It should be noted that the obtained results based on these methods can not be representative of a whole reach subject to disequilibrium.

Continuous channel adjustments have occurred in the Jingjiang Reach of the Middle Yangtze River, because of the operation of the Three Gorges Project (TGP) and the construction of various river regulation works along the reach^11^,^12^,^14^,^20^,^21^,^22^,^23^. Therefore, it is important to investigate the recent dynamic adjustments in the reach-scale bankfull channel geometry, for better understanding of the channel evolution of this reach. The aims of the current study are to: (i) present the altered flow and sediment regime and the process of channel degradation in the study reach owing to the TGP operation; (ii) calculate the reach-scale bankfull channel dimensions based on an improved reach-averaged method and surveyed cross-sectional profiles; and (iii) develop empirical relationships between these bankfull dimensions and the corresponding incoming flow and sediment regime.

## Study Area

**Figure 1:**
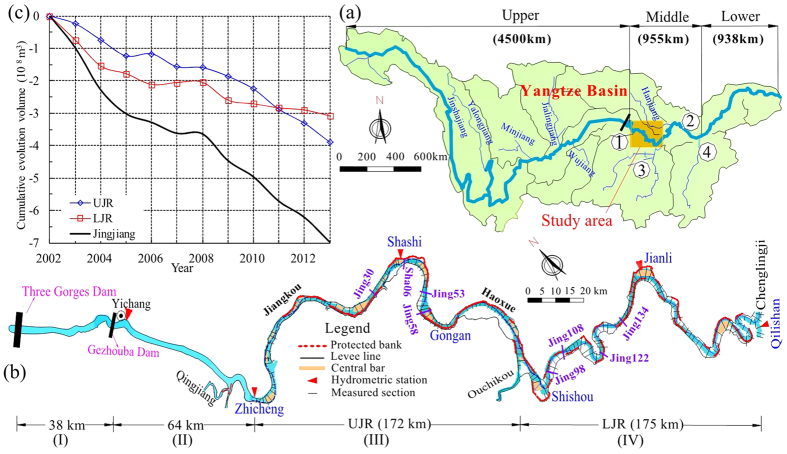
(**a**) Yangtze River Basin. ①-Yichang; ②-Hukou; ③-Dongting Lake; ④- Poyang Lake; (**b**) Sketch of Jingjiang Reach with the locations of 171 cross sections and hydrometric sections. The Gezhouba Water Conservancy Project is located 38 km downstream of the TGD, and this project is the first large-scale run-of-river hydropower station on the Yangtze River with low-head and high flow. The cumulative volumes of channel scour in the sub-reaches I–IV were 0.41 × 10^8^, 1.44 × 10^8^, 3.9 × 10^8^ and 3.1 × 10^8^ m^3^ in 2002–2013; and (**c**) Cumulative channel evolution volume in the Jingjiang Reach since 2002, with the negative value meaning channel scour. With the TGP operation, the UJR and LJR generally underwent continuous channel degradation owing to the reduced sediment load, and slight channel aggradation occurred in a few years.

The Yangtze River, with a total length of 6300 km, usually is divided into upper, middle and lower reaches according to different geomorphological environment and hydrological characteristics (Fig. 1a). The Middle Yangtze River lying between Yichang and Hukou has a length of 955 km, covering the lakes of Dongting and Poyang^24^,^25^. Runoff and sediment in the Jingjiang Reach come from the main stream and tributaries in the Upper Yangtze River, and the majority of the water volume and sediment load are transported intensively during flood seasons from May to October. The Three Gorges Dam (TGD) is located at the exit of the Upper Yangtze River (Fig. 1b).

The Jingjiang Reach is located between Zhicheng and Chenglingji in the Middle Yangtze River, about 102 km downstream of the TGD (Fig. 1b), and there are three diversion branches linking the Middle Yangtze River with the Dongting Lake. The branches usually divert water from the main stream during flood seasons but are normally dry during non-flood seasons. Due to the differences in the flow and sediment regime, the composition of bed and bank materials and the channel pattern, the total Jingjiang Reach is usually divided into the Upper Jingjiang Reach (UJR) with a length of 172 km and the Lower Jingjiang Reach (LJR) with a length of 175 km, marking the boundary at one diversion inlet of Ouchikou^24^.

The Upper Jingjiang Reach is a slightly curved and multi-branched channel, consisting of six river bends, with central bars distributed widely in these bends. The river channel upstream of Jiangkou is controlled mainly by low hills and stable riverbanks, and the surface layer of the bed is mainly composed of sand and gravel; the river channel downstream of Jiangkou is located on an alluvial plain, with the riverbanks being composed of a thin sand lower layer and a thick clay upper layer. The Lower Jingjiang Reach is a typical meandering channel, consisting of ten river bends. The bed material in the LJR channel comprises medium-fine sand, and the majority of the riverbanks consist of a typical two-layer structure, with a thick non-cohesive lower bank and a thin cohesive upper bank of just a few metres in total. In order to prevent extreme floods, levees have been constructed along both sides of the Jingjiang Reach (Fig. 1b)^25^,^26^. In the UJR, most critical zones for bank erosion with the main stream approaching or impinging have been defended by various bank protection works, and approximately 120 km of the UJR is now protected by channel stabilization works to prevent local bank erosion. In the LJR, riverbanks on both sides generally have been protected by the construction of revetment engineering for more than half a century, and about 146 km of the LJR is now protected by various bank revetment engineering. Therefore, less than 40% of the banklines in the Jingjiang Reach have been protected by bank protection works.

In order to accurately monitor the processes of recent channel evolution, 171 cross-sections at specified locations were established along the reach by the Changjiang Water Resources Commission (CWRC)^25^,^27^, and post-flood surveys of cross-sectional profiles at these locations have been conducted annually since 2002 (Fig. 1b). The number of measured cross-sections is 96 in the UJR and 75 in the LJR. The distance between two consecutive sections ranges between 0.48 and 5.53 km, with a mean spacing of about 2 km. However, the spacing is refined in complex regions, such as sharply curved or bifurcated reaches. In addition, these sections are usually located nearly perpendicular to the main stream of the channel. Such a distribution of cross-sections along the reach seeks to represent the variation of channel pattern and to allow an accurate calculation of the volume of bed deformation^25^,^27^.

### Variation in flow and sediment regime

**Figure 2:**
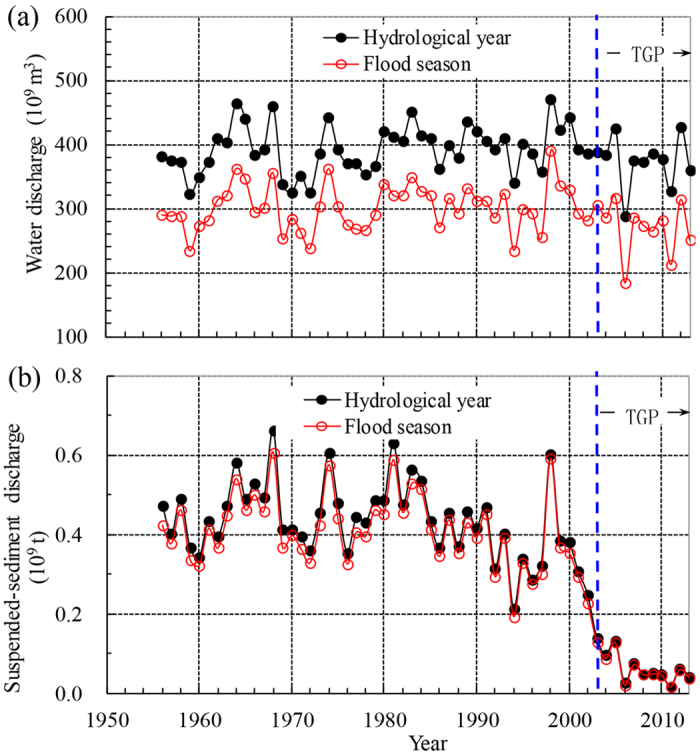
Temporal variations in the flow and sediment regime entering the Jingjiang Reach of (**a**) Annual and flood-season water discharges; and (**b**) Annual and flood-season suspended-sediment discharges. All the hydrological data from the Bureau of Hydrology, Changjiang Water Resources Commission.

Since the TGP operation in June 2003, the sediment load entering the Jingjiang Reach has been drastically reduced, and the channel is therefore undergoing continuous degradation because of the flows with low sediment concentrations released from the reservoir^12^,^25^. In order to investigate the adjustment characteristics in the bankfull channel geometry, recent hydrological data in the study reach were collected from the CWRC, including the average daily discharges and sediment concentrations at the hydrometric stations of Shashi and Jianli. These hydrological data show that the mean discharge at Shashi was about 17048 m^3^/s after the TGP operation from 2002 to 2013 (Fig. 2a), and the corresponding mean concentration of suspended sediment was 0.28 kg/m^3^, less than the mean value of 1.35 kg/m^3^ from 1956 to 2002 (Fig. 2b). The TGP usually adopts a regulation mode of flood control by reducing the peak discharge when the incoming discharge upstream exceeds a critical value during a flood season, and the maximum discharge released is regulated to be less than 40000 m^3^/s. Therefore, the peak discharges have reduced significantly, which leads to lower average monthly discharges during flood seasons^25^. In addition, the average monthly discharges have increased slightly during non-flood seasons, with a higher occurrence probability for the released moderate discharges ranging between 15000 and 25000 m^3^/s.

### Channel evolution in the Jingjiang Reach

Various erosion and deposition processes occurred in the Jingjiang Reach before the TGP operation^12^,^14^,^20^,^23^,^25^,^27^. For example, the channel experienced continuous degradation after three cut-offs of the LJR during the period from 1966 to 1980, with a cumulative channel-scour volume of 3.46 × 10^8^ m^3^ below the bankfull level^25^,^27^. The channel degradation continued after the completion of the Gezhouba Project, with a cumulative channel-scour volume of 1.29 × 10^8^ m^3^ in 1980–1986, and with a cumulative volume of channel scour of 0.19 × 10^8^ m^3^ between 1986 and 2002^25^,^27^. Channel bathymetry measured in 1993 indicates that the mean depths corresponding to the bankfull level were 11.2 m and 10.4 m in the UJR and LJR, respectively, with the corresponding ratios of width to depth being equal to 133.9 and 133.7^24^. In general, the channel in the study reach had a relatively wide and shallow geometry before the TGP operation.

#### Recent channel degradation process

Calculations based on these repeated surveys of cross-sections indicate that the cumulative volume of channel scour corresponding to the bankfull level reached 7.0 × 10^8^ m^3^ in the whole reach from 2002 to 2013, with the values of 3.9 × 10^8^ m^3^ in the UJR and 3.1 × 10^8^ m^3^ in the LJR, respectively (Fig. 1c)^27^. The UJR appeared to undergo less degradation than the LJR until 2011, while the rate of channel scour in the UJR was greater than that in the LJR after 2011. The average annual rate of channel scour in the Jingjiang Reach was 0.636 × 10^8^ m^3^/yr after the TGP operation, which was lower than the previous model predictions varying between 0.78 and 1.19  × 10^8^ m^3^/yr, presented by different research institutes^28^. However, such a rate of channel scour was much greater than the average annual scour rate during the period from 1980 to 2002 before the TGP operation (0.067 × 10^8^ m^3^/yr).

The special geomorphological environment and hydrological characteristics contribute to such channel evolution characteristics in the reach. The cumulative volume of channel scour in the reach between TGD and Zhicheng was 1.85 × 10^8^ m^3^ over the same period (Fig. 1b), covering the cumulative volume of 0.41 × 10^8^ m^3^ between TGD and Gezhouba Dam, and the volume of 1.44 × 10^8^ m^3^ between Gezhouba Dam and Zhicheng^27^. Therefore, the channel evolution processes upstream of Zhicheng had a limited influence on the channel adjustments in the Jingjiang Reach. Previous studies indicate that the river regime in the Jingjiang Reach generally remained stable after the TGP operation, with no transition in river pattern being identified^25^,^28^.

#### Variation in typical cross-sectional profiles

**Figure 3:**
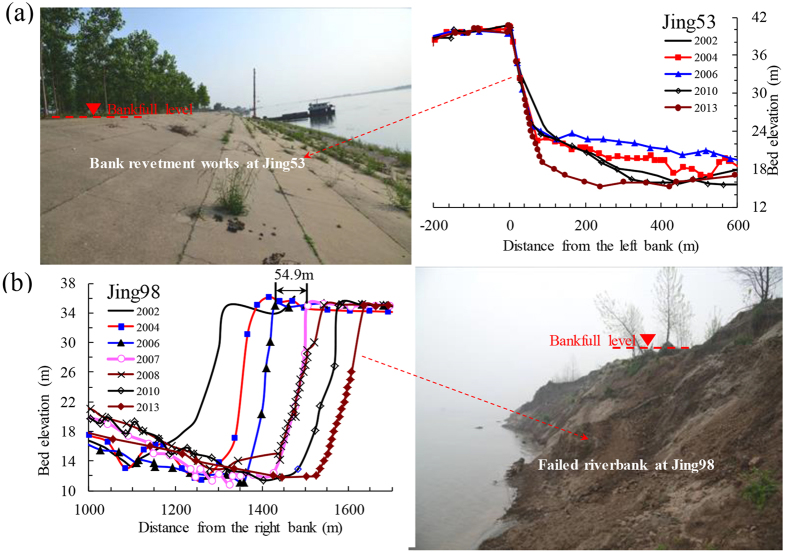
Temporal changes of bed and bank profiles at two sections of (**a**) Jing53 in the UJR, which is located about 15.1 km downstream of Shashi; and (**b**) Jing98 in the LJR, which is located about 57.6 km upstream of Jianli. Changes in the bed and bank profiles at these sections were typical of the whole study reach, including stable and erodible banks. The variations in typical cross-sectional profiles were caused partly by the downstream movement of sand waves due to the transport of sand-bed load, and mainly by the non-equilibrium transport of suspended load. The intra-annual variability in the channel evolution shows an alternating process of erosion and deposition over a hydrological year, with a general trend in channel scour.

The temporal changes of bed and bank profiles at Jing53 in the UJR and at Jing98 in the LJR are shown in Fig. 3. The left riverbank of Jing53 remained stable, although there was a considerable process of bed evolution in the near-bank region (Fig. 3a). At the right riverbank of Jing98 without the protection of bank revetment engineering, the cumulative bank-retreat width reached 321 m during the period from 2002 to 2013, with a maximum bank retreat rate of 54.9 m/yr in 2007 (Fig. 3b). Such a high bank erosion rate was typical in a local reach with erodible banks. Therefore, serious bank erosion processes occurred at local reaches without sufficient bank revetment works because of the recent channel degradation. However, in a local reach protected by bank revetment works, a section usually had a stable left or right bank, with the dominated characteristic of channel incision. According to incomplete statistics available, the average numbers of cross-sections undergoing significant bank erosion processes were 12 and 18 in the UJR and LJR, respectively, and therefore, the intensity of bank erosion in the LJR was greater than that in the UJR. However, the channel planform in the study reach generally remained stable because of the effect of various bank protection engineering.

#### Adjustments in other fluvial factors

The TGP altered the flow and sediment regime entering the Jingjiang Reach significantly, with the sediment concentrations reducing greatly. Therefore, a process of clear-water scouring occurred along the study reach, which led to adjustments in other fluvial factors, such as the longitudinal channel slope and bed material composition^25^,^29^,^30^,^31^. The longitudinal channel profile was represented by the connection curve of mean main-channel elevations at all the sections included in the study reach. With these measured profiles, the mean longitudinal channel slopes were calculated annually over the years from 2002 to 2013, using a simple method of linear regression. These calculations indicate that the slopes of the study reach tended to flatten gradually with the channel degradation, decreasing from 0.048 × 10^−3^ in 2002 to 0.044 × 10^−3^ in 2013 (Supplementary Fig. S1a). Measurements of the bed material composition indicate that the medium diameter of the bed material in the UJR increased from 0.202 mm in 2001 to 0.269 mm in 2012, with the corresponding value in the LJR increasing from 0.167 to 0.212 mm (Supplementary Fig. S1b)

### Calculated reach-scale bankfull channel dimensions

**Figure 4:**
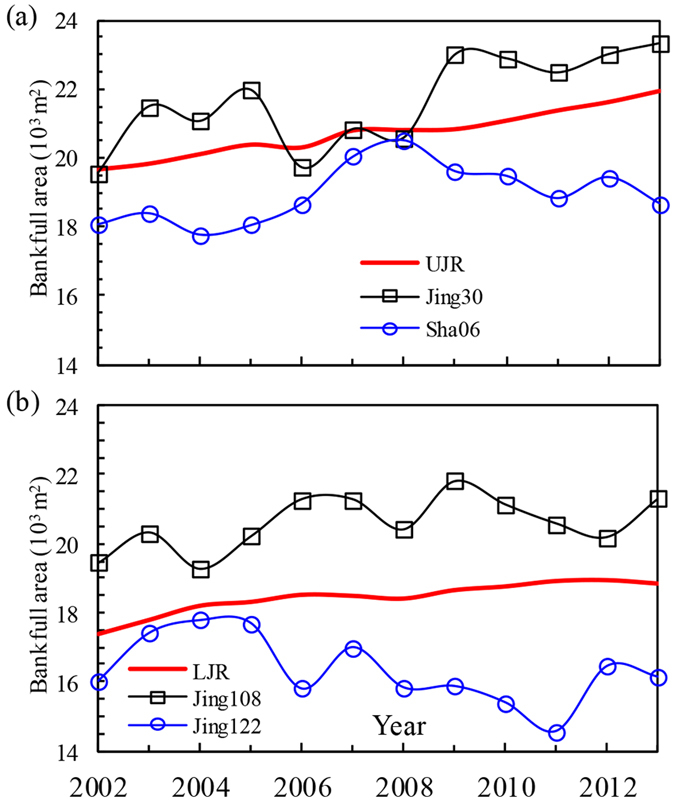
Temporal variations in the reach-scale (heavy lines) and section-scale (thin lines with data marks) cross-sectional areas of (**a**) Upper Jingjiang Reach and (**b**) Lower Jingjiang Reach, showing the progression of channel scour in the Jingjiang Reach during the period from 2002 to 2013. In the UJR, Jing30 section is located 14.4 km upstream of Shashi, and Sha06 section is located 1.4 km downstream of Shashi; in the LJR, Jing108 and Jing122 sections are located 47.1 and 29.9 km upstream of Jianli, respectively. Jing30 is also located at the front of the central bar of Taipingkou. As shown in Table 1, the flood-season average discharge in 2006 was relatively low (11568 m^3^/s), with obvious sediment deposition occurring at Jing30, which led to a sharp reduction in the bankfull area in 2006. During the period from 2007 to 2009, this central bar was eroded significantly due to large discharges during flood seasons, which made a remarkable contribution to an increase in the bankfull area at Jing30.

Using the reach-averaged calculation procedure, the collected post-flood profiles at 171 sections in the study reach from 2002 to 2013 firstly were used to determine the section-scale bankfull channel geometry, and the reach-scale bankfull width 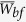, depth 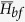 and area 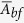 were then calculated separately for the UJR and LJR because of a slight difference in channel pattern (Fig. 4 and Table 1). With the continuous channel degradation, the reach-averaged bankfull channel geometry in the Jingjiang Reach adjusted gradually over the period from 2002 to 2013, which was characterised by a prominent increase in bankfull depth (Table 1), because of the effective restriction in bankfull width adjustment caused by various bank revetment works. The reach-scale bankfull widths in the UJR and LJR changed slightly from 2002 to 2013, with the mean widths in these reaches of 1388 and 1305 m, respectively. The reach-scale bankfull depth increased from 14.2 m in 2002 to 15.8 m in 2013, with an increase of 1.6 m in the UJR, while it increased from 13.5 m in 2002 to 14.5 m in 2013, with an increase of 1.0 m in the LJR (Table 1). As compared with the pre-TGP bankfull channel geometry measured in 1993, the Jingjiang Reach tended to have a relatively narrow and deep channel geometry owing to the TGP operation, with the bankfull width to depth ratios in two reaches respectively reducing to 87.6 and 88.7 in 2013. It should be noted that: the adjustments in the bankfull channel geometry of the study reach differed significantly from those adjustments occurring recently in a braided reach of the Lower Yellow River in response to the operation of Xiaolangdi Reservoir; and the reach-scale bankfull width in the braided reach increased by about 390 m in 1999–2012 because of imperfect river training works and floodplain protection works, with an increase in the bankfull depth of 1.8 m over the same period^9^.

The temporal variations in the reach-scale bankfull area, as well as the section-scale bankfull areas at Jing30 and Sha06 in the UJR are shown in Fig. 4a. The bankfull area at Jing30 varied from 19548 m^2^ in 2002 to 23324 m^2^ in 2013, with an increase of 19.3% over the period. The bankfull area at Sha06 had an increase of 3.4% over the past 11 years. Therefore, the bankfull areas at these two sections had a greater variation range than that of the reach-scale values in the UJR. The temporal variations in the reach-scale bankfull area and the bankfull areas at Jing108 and Jing122 in the LJR are shown in Fig. 4b. The mean bankfull area at Jing108 was greater than that at Jing122, but the variation range of the bankfull area at each section was relatively greater than that of the reach-scale values in the LJR. Therefore, Fig. 4 represents temporal variation trends of the bankfull areas at four different sections. However, the reach-scale bankfull areas in the UJR and LJR demonstrated a gradual increasing trend with the channel degradation. In addition, the bankfull cross-sectional areas differed significantly along the reach, and these cross-sectional areas measured in 2002 ranged from 10772 to 31314 m^2^ due to the variability in the bankfull width and depth (Supplementary Fig. S2). Such temporal and spatial distributions of the bankfull dimensions are caused by the longitudinal differences in the channel morphology, the composition of bed and bank materials, and river regulation works.

### Dynamic adjustments in the channel geometry

The dynamic adjustments in the bankfull channel dimensions of the Jingjiang Reach have been influenced significantly by recent human activities, including upstream damming and large-scale river training works along the reach. The average annual sediment load at Shashi was 0.823 × 10^8^ t/yr in 2002–2013 (Fig. 2b), with the corresponding mean sediment load during flood seasons of 0.795 × 10^8^ t/yr; and the sediment load entering the reach was transported mainly during flood seasons because of the operation of the TGP. Therefore, the channel evolution in the reach mainly occurred during the flood seasons, and the intensity of channel evolution during the non-flood seasons was negligible as compared with that during the flood seasons. According to the analysis of observed data by the CWRC^27^, the average ratio of sand-bed load to suspended load at Zhicheng in the Jingjiang Reach was less than 4% over the period from 2003 to 2013. Therefore, the effect of bed-load transport on the channel evolution usually was ignored, with the transport of suspended load only being considered in the current study.

**Figure 5:**
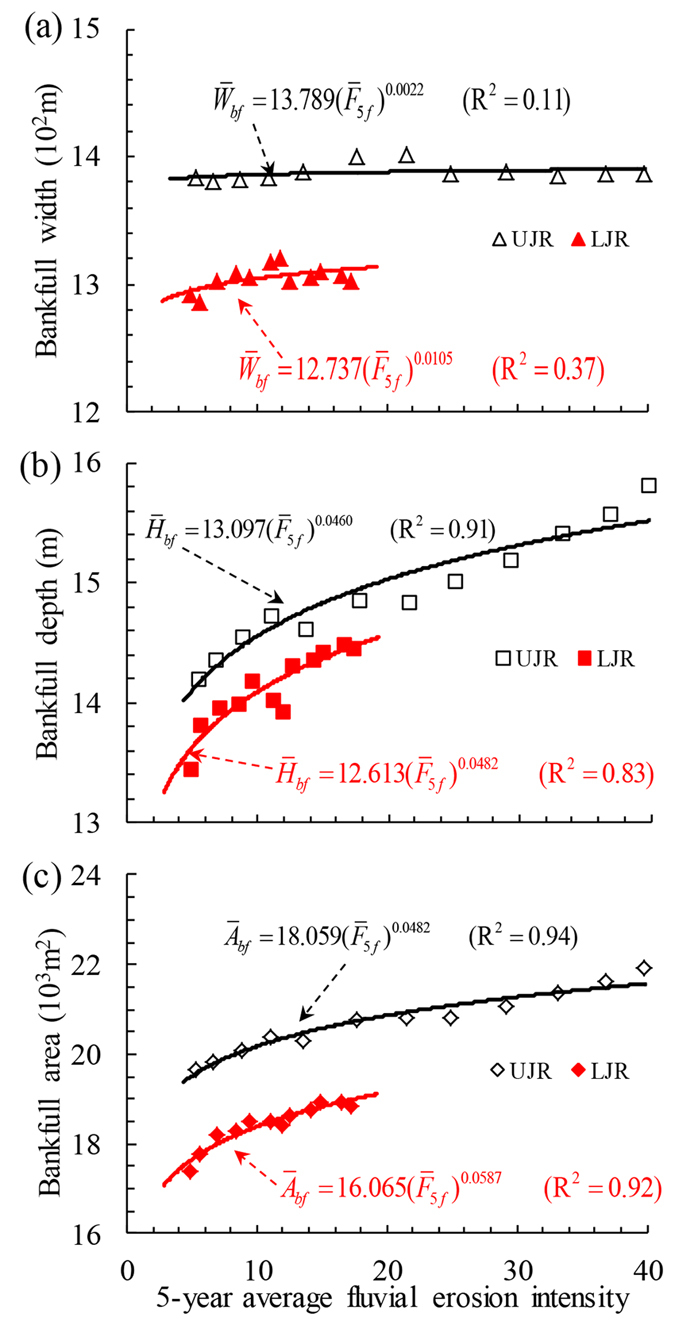
Relationships between the reach-scale bankfull dimensions and the five-year average fluvial erosion intensity during flood seasons in the UJR and LJR: (**a**) Bankfull width; (**b**) Bankfull depth; and (**c**) Bankfull area. The average reach-scale bankfull width or depth in the UJR was greater than the corresponding value in the LJR, and such a distribution of hydraulic-geometry parameters in the Jingjiang Reach is caused by the particular geomorphological environment and hydrological characteristics.

The correlations (R^2^) between each reach-scale channel dimension (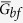) and mean fluvial erosion intensity during flood seasons were tested for different moving average years, and it was found that R^2^ had the highest value for the previous three to seven years in the study reach. Therefore, generally it is concluded that the variation in the bankfull channel geometry of the UJR or LJR responded well to the previous 5-year average fluvial erosion intensity during flood seasons (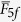). The relationships between 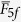 and 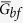 in the UJR and LJR are shown in Fig. 5. We can find from Fig. 5a that: (i) the values of 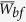
remained almost unchanged for different values of 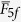 in the UJR, with the correlation between them being very weak (R^2^ = 0.11), because of the effects of the large-scale river training works and the thick clay upper riverbanks; and (ii) these values slightly increased for a higher 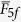 in the LJR, with a relatively higher correlation of R^2^ = 0.37, which means that the adjustment in bankfull width of local reaches in the LJR was relatively remarkable due to the insufficient bank revetment works and the thin clay upper riverbanks. However, there existed a strong correlation between 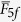 and 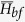 in each reach (Fig. 5b), with the correlation coefficients of 0.91 and 0.83 in the UJR and LJR, respectively, which indicates that the reach-scale bankfull depth has generally adjusted to the altered flow and sediment regime caused by the TGP operation. As a comprehensive representative parameter in bankfull channel geometry, 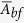 in the UJR or LJR correlated well with the corresponding parameter 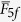
(Fig. 5c). Therefore, the recent dynamic adjustments in the bankfull channel geometry of the Jingjiang Reach mainly were represented by the variation in bankfull depth, because the variation in bankfull width was significantly restricted by bank revetment works along the reach.

## Conclusions

Because of the recent TGP operation, the flow and sediment regime entering the Jingjiang Reach in the Middle Yangtze River has been altered significantly, with the magnitude of sediment load being reduced drastically. The mean concentration of suspended sediment at Shaishi decreased from 1.35 kg/m^3^ in 1956–2002 to 0.28 kg/m^3^ in 2002–2013, which led to considerable channel evolution of the study reach. Continuous channel degradation caused a cumulative channel scour volume of 7.0 × 10^8^ m^3^ in 2002–2013, and the average annual rate of channel scour was 0.636 × 10^8^ m^3^/yr after the TGP operation (Fig. 1c), which was much greater than the pre-TGP average annual scour rate of 0.067 × 10^8^ m^3^/yr in 1980–2002. Calculated reach-scale bankfull channel dimensions in the UJR and LJR indicate that the channel adjustments mainly occurred in the component of bankfull depth, with the increased depths of 1.6 m in the UJR and 1.0 m in the LJR, respectively. These changes led to a relatively narrow and deep channel geometry (Table 1). Empirical relationships were developed between the reach-scale bankfull channel dimensions and the previous five-year average fluvial erosion intensity during flood seasons in two reaches, with higher correlations being obtained for the reach-scale bankfull depth and area (Fig. 5). It is concluded that: the adjustments in the bankfull channel geometry of the UJR and LJR mainly resulted from recent human activities such as the TGP operation; the bankfull widths in the UJR and LJR changed slightly owing to the effect of various bank revetment works, with the bankfull depths demonstrating a gradual increasing trend with the channel degradation; and the river regime and channel planform of the study reach generally remained stable.

## Methods

### Calculation procedure for reach-scale bankfull channel geometry

Measured cross-sectional profiles in the Jingjiang Reach indicate significant longitudinal variability in the section-scale bankfull channel geometry (Supplementary Fig. S2). It is appropriate to adopt a reach-averaged method to calculate the bankfull channel geometry in the reach. The reach-averaged method of Harman *et al.*^3^ was improved, with the calculation procedure being outlined briefly^9^.

The first step is to determine the bankfull channel dimensions at a section with a specified bankfull level and main-channel zone. In terms of flood control, the level of the edge of an active floodplain is usually defined as the bankfull level at a section (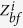), and the passage between two edges of the active floodplains on both sides is often defined as the zone of the main channel, with the corresponding mean bed elevation being expressed by 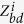. Surveyed cross-sectional profiles indicate that the sections in the UJR usually have an asymmetric W-shaped profile because of the presence of central bars (Supplementary Fig. S3a); and the sections in the LJR are usually characterised by a V-shaped profile (Supplementary Fig. S3b). Therefore, it is relatively easy to determine the bankfull dimensions at a section. The second step is to calculate the reach-scale bankfull channel dimensions using the reach-averaged method, which integrates a geometric mean based on the log-transformation with a weighted average based on the spacing between two consecutive sections^9^. The reach-averaged method assumes that the study reach with a channel length of *L* covers a number of cross-sections, and the bankfull channel dimensions at the *i*th section need to be determined by the first step, including the bankfull width (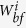), depth (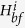), and cross-sectional area (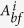), where the mean bankfull channel depth (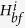) is equal to the ratio of 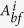 to 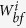. Therefore, the mean bed elevation of main channel (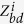) is equal to 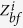 − 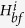. With the integrated method, the corresponding reach-scale bankfull geometry (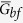) can be written as:


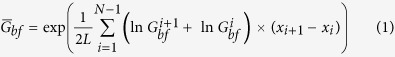


where 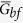 represents one of the reach-scale bankfull width (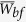), depth (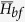) and area (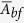); 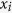 is the longitudinal distance at the *i*th section downstream of the dam; and *N* is the number of cross-sections included in the reach. This equation indicates that the calculated reach-scale bankfull geometry can guarantee the continuity of channel dimensions, which means that 
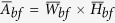
 always holds true using this method. In addition, this approach can also account for the effect of varied spacing between two sections on the reach-scale bankfull parameters.

### Comprehensive parameter for the flow and sediment regime

Existing studies indicate that the value of the bankfull channel geometry (for example, bankfull width and depth) is an empirical function of previous years’ flow and sediment conditions during flood seasons, and these conditions are usually represented by the previous *n* years’ average discharge and incoming sediment coefficient during flood seasons^7^,^9^. The incoming sediment coefficient (*ξ*) is expressed by a ratio of the mean suspended-sediment concentration (*S*) to the corresponding mean discharge (*Q*) during a flood season, and a power function of *Q* and *ξ* is usually used to represent the fluvial erosion intensity in an alluvial river carrying high sediment concentrations. In the Jingjiang Reach carrying low sediment concentrations, the mean fluvial erosion intensity during a flood season (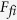) usually is used to represent the incoming flow and sediment regime for the *i*th year^32^, which is defined as:


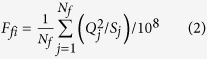


where 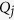 and 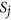 are the average daily discharge [m^3^/s] and suspended-sediment concentration [kg/m^3^], respectively; and 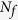 is the number of the total days covered in the flood season. In an alluvial river reach under quasi-equilibrium, there is an empirical relationship between sediment discharge (*Q*_*S*_) and water discharge (*Q*) at a hydrometric station, and this relation can be written usually as: *Q*_*S*_ = *a* (Q)^*b*^, where *a* is a coefficient, and *b* is an exponent. Based on the hydrological data measured at Shashi and Jianli before 2002, the parameter *b* was calibrated to be around 2.0. Therefore, 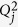 in Eq. (2) approximately represents the sediment transport capacity at a given section, and the term 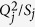 in Eq. (2) is regarded as a comprehensive parameter, which represents the ratio of sediment transport capacity to incoming sediment concentration under a given discharge. In general, Eq. (2) only presents an empirical expression for the fluvial erosion intensity, with non-homogeneous dimension.

### Delayed response equation of reach-scale bankfull dimensions to human activities

The following analysis confirms that the variation in the bankfull channel dimensions in the Jingjiang Reach can respond well to the previous 5-year average fluvial erosion intensity during flood seasons (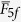), which can be expressed by 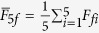. Because of a difference in the flow and sediment regime between the LJR and UJR, the calculated values of 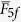 in the UJR were based on the hydrological data at Shashi, whereas these values in the LJR were based on the hydrological data at Jianli.

The dynamic adjustments in the bankfull channel geometry of the Jingjiang Reach were usually characterised by two main components of bankfull width and depth, with their product equalling the magnitude of bankfull area. Further analysis also shows the reach-scale bankfull geometry 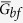 (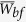, 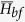 and 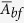) is closely related to the parameter 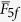. Therefore, a relation for predicting the value of 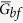 in the UJR or LJR can be expressed by


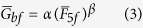


where *α* is a coefficient; and *β* is an exponent. With the calculated values of 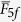 and 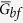 from 2002 to 2013, the parameters *α* and *β* in Eq. (3) were calibrated for each reach using the log-transformation and linear regression analysis (Supplementary Tab. S1).

## Additional Information

**How to cite this article**: Xia, J. *et al.* Dynamic channel adjustments in the Jingjiang Reach of the Middle Yangtze River. *Sci. Rep.* **6**, 22802; doi: 10.1038/srep22802 (2016).

## Supplementary Material

Supplementary Information

## Figures and Tables

**Table 1:** Reach-scale bankfull widths and depths in different reaches with incoming discharges and suspended-sediment concentrations during flood seasons.

Year	Upper Jingjiang Reach					Lower Jingjiang Reach				
	*Q*_*UJR*_	*S*_*UJR*_	*F*_*UJR*_	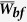	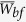 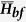	*Q*_*LJR*_	*S*_*LJR*_	*F*_*LJR*_	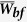	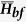
	(m^3^/s)	(kg/m^3^)		(m)	(m)	(m^3^/s)	(kg/m^3^)		(m)	(m)
2002	17657	0.81	5.16	1384	14.20	16190	0.71	4.52	1292	13.45
2003	19185	0.42	12.34	1381	14.36	17619	0.42	9.91	1287	13.82
2004	17944	0.30	15.81	1382	14.55	17148	0.33	11.91	1303	13.97
2005	19919	0.40	16.53	1384	14.73	18951	0.43	11.99	1308	14.00
2006	11568	0.11	18.29	1388	14.62	11236	0.18	8.90	1306	14.18
2007	17940	0.25	25.75	1400	14.85	17298	0.31	12.71	1317	14.03
2008	17201	0.17	31.22	1402	14.84	16526	0.24	13.85	1321	13.93
2009	16660	0.18	32.91	1386	15.03	16293	0.24	15.20	1303	14.31
2010	17690	0.16	37.80	1388	15.19	16786	0.20	20.19	1306	14.36
2011	13371	0.07	38.16	1386	15.42	13101	0.15	12.68	1311	14.43
2012	19705	0.19	44.19	1387	15.58	18632	0.23	20.55	1307	14.50
2013	15738	0.15	45.88	1386	15.82	15235	0.21	17.86	1303	14.45
Mean	17048	0.28	27.00	1388	14.93	16251	0.31	13.36	1305	14.12

The suspended-sediment concentrations at Jianli were consistently greater that the values at Shashi after the TGP operation, and the increase of suspended load along the reach was caused by the recent channel scour of the reach below Shashi. There is a diversion branch at Ouchikou located in the reach between Shashi and Jianli, which led to the water discharges at Shashi being consistently greater than the values at Jianli. The ratio of the annual water discharge entering three diversion branches to the discharge at Zhicheng decreased gradually, with the average ratio reducing from 20.1% before 2002 to 11.7% in 2003–2013^27^. A decrease in the diversion ratio of flow means that fewer flood-season discharges were diverted from the main stream of the Yangtze Reach into the Dongting Lake.

*Q*_*UJR*_, *S*_*UJR*_ = mean discharge and suspended-sediment concentration at Shashi during a flood season from May to October.

*Q*_*LJR*_, *S*_*LJR*_ = flood-season average discharge and suspended-sediment concentration at Jianli.

*F*_*UJR*_, *F*_*LJR*_ = flood-season average fluvial erosion intensity at Shashi and Jianli.

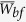
, 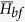 = reach-scale bankfull width and depth.
