# Supplementary material for: Dynamic channel adjustments in the Jingjiang Reach of the Middle Yangtze River
Source: Sci Rep. 2016 Mar 11;6:22802. doi: 10.1038/srep22802 (PMC4786812; doi:10.1038/srep22802)
Supplement: Supplementary Information [file srep22802-s1.pdf]

Dynamic channel adjustments in the Jingjiang Reach of the Middle Yangtze River

Junqiang Xia[1*], Shanshan Deng[1], Jinyou Lu[2], Quanxi Xu[3], Quanli Zong[1], and Guangming Tan[1]

[1] *State Key Laboratory of Water Resources and Hydropower Engineering Science, Wuhan University, Wuhan 430072, China; Email: xiajq@whu.edu.cn*

[2] *Changjiang River Scientific Research Institute, Wuhan 430010, China*

[3] *Bureau of Hydrology, Changjiang Water Resources Commission, Wuhan 430010, China*

Supplementary information:

Figure S1 (temporal variations in other fluvial factors);

Figure S2 (longitudinal variation in the bankfull channel geometry measured in 2002);

Figure S3 (calculated bankfull channel dimensions at two sections); and

Table S1 (calculated parameters in Eq. (3) for the UJR and LJR)

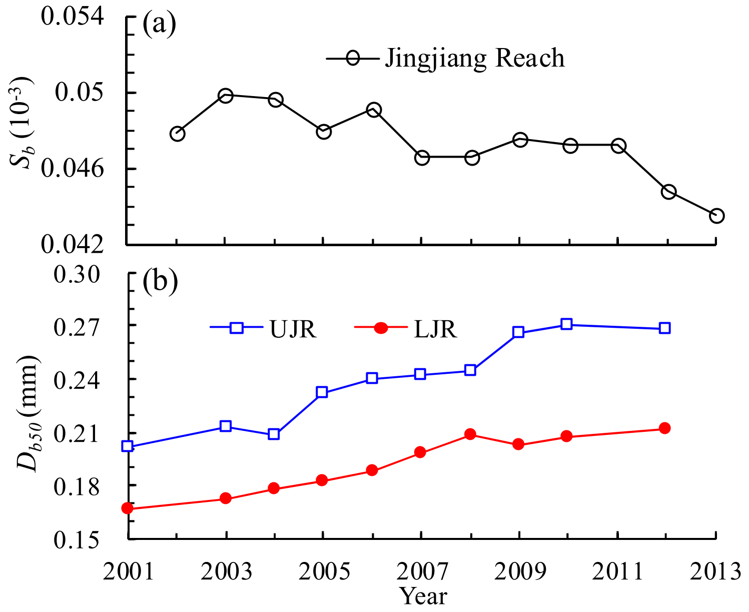

Figure S1 Temporal variations in other fluvial factors of (a) longitudinal channel slope (S_b), and (b) medium diameters of the bed material (D_{50}) in the UJR and LJR.

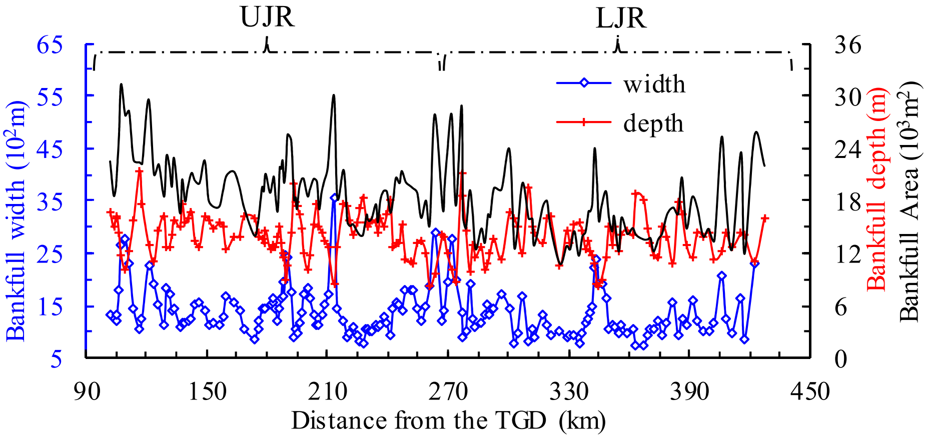

Figure S2. Longitudinal variation in the bankfull channel geometry measured in 2002. The bankfull channel widths changed considerably along the reach, and there was a minimum W_{bf} of <800 m and a maximum W_{bf} of about 3600 m in the UJR, and they ranged from 750 to 2900 m in the LJR. The bankfull depths also varied significantly along the reach, with a variation range of 8 to 21 m. Due to the variability in the bankfull channel width and depth, the bankfull cross-sectional areas changed greatly along the reach, ranging from 10772 to 31314 m^2.

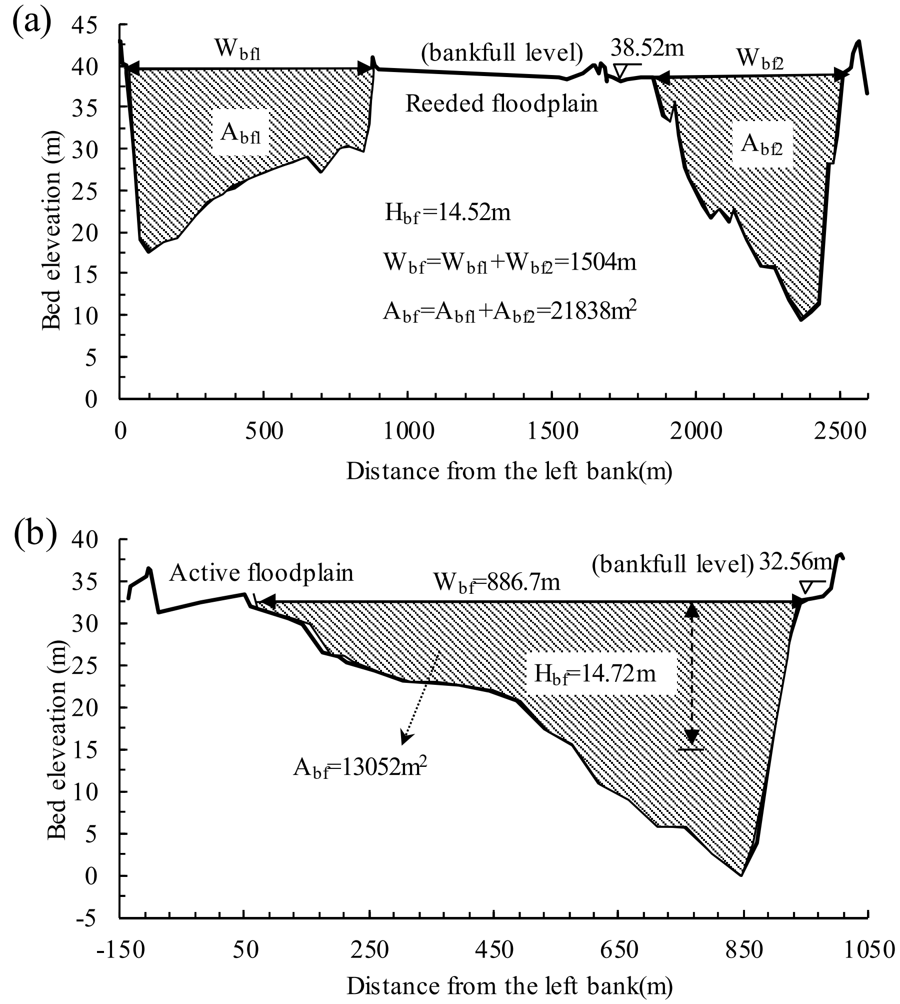

Figure S3. Calculated bankfull channel dimensions at two sections of (a) Jing58 in the UJR (the determined bankfull level at Jing58 was 38.52 m in 2012, with a total bankfull width and area for the two branches of 1504 m and 21838 m^2, respectively), and (b) Jing134 in the LJR (the bankfull area at Jing134 in 2012 was 13052 m^2 under a bankfull level of 32.56 m). The original floodplains remain active due to a limited incision depth during the recent channel evolution, and the level of the edge of an active floodplain is usually defined as the bankfull level at a section.

Table S1. Calculated parameters in Eq. (3) for the UJR and LJR.

Reaches	Bankfull channel geometry	Parameters α	Parameters β	R^2	Number of sections
UJR	$\bar{W}_{bf}$	1378.9	0.0022	0.11	96
	$\bar{H}_{bf}$	13.097	0.0460	0.91	
	$\bar{A}_{bf}$	18059	0.0482	0.94	
LJR	$\bar{W}_{bf}$	1273.7	0.0105	0.37	75
	$\bar{H}_{bf}$	12.613	0.0482	0.83	
	$\bar{A}_{bf}$	16065	0.0587	0.92	
